# Genome analysis of a simultaneously predatory and prey-independent, novel *Bdellovibrio bacteriovorus* from the River Tiber, supports *in silico* predictions of both ancient and recent lateral gene transfer from diverse bacteria

**DOI:** 10.1186/1471-2164-13-670

**Published:** 2012-11-27

**Authors:** Laura Hobley, Thomas R Lerner, Laura E Williams, Carey Lambert, Rob Till, David S Milner, Sarah M Basford, Michael J Capeness, Andrew K Fenton, Robert J Atterbury, Maximilian ATS Harris, R Elizabeth Sockett

**Affiliations:** 1 Centre for Genetics and Genomics, School of Biology, University of Nottingham, Medical School QMC, Derby Road, Nottingham, NG7 2UH, UK; 2 Institute for Genome Sciences and Policy, Duke University, Durham, NC, 27708, USA; 3 Current addresses: College of Life Sciences, University of Dundee, Dow Street, Dundee, Scotland, DD1 5EH, UK; 4 Current addresses: Division of Mycobacterial Research, MRC National Institute for Medical Research, The Ridgeway, Mill Hill, London, NW7 1AA, UK; 5 Current addresses: Centre for Bacterial Cell Biology, University of Newcastle, Newcastle Upon Tyne, NE2 4AX, UK; 6 School of Veterinary Medicine & Science, University of Nottingham, Sutton Bonington Campus, Leicestershire, LE12 5RD, UK

**Keywords:** Evolutionary genetics of bacteria, Lateral gene transfer, Predatory *Bdellovibrio*

## Abstract

**Background:**

Evolution equipped *Bdellovibrio bacteriovorus* predatory bacteria to invade other bacteria, digesting and replicating, sealed within them thus preventing nutrient-sharing with organisms in the surrounding environment. *Bdellovibrio* were previously described as “obligate predators” because only by mutations, often in gene *bd0108,* are 1 in ~1x10^7^ of predatory lab strains of *Bdellovibrio* converted to prey-independent growth. A previous genomic analysis of *B. bacteriovorus* strain HD100 suggested that predatory consumption of prey DNA by lytic enzymes made *Bdellovibrio* less likely than other bacteria to acquire DNA by lateral gene transfer (LGT). However the Doolittle and Pan groups predicted, *in silico*, both ancient and recent lateral gene transfer into the *B. bacteriovorus* HD100 genome.

**Results:**

To test these predictions, we isolated a predatory bacterium from the River Tiber- a good potential source of LGT as it is rich in diverse bacteria and organic pollutants- by enrichment culturing with *E. coli* prey cells. The isolate was identified as *B. bacteriovorus* and named as strain Tiberius. Unusually, this Tiberius strain showed *simultaneous* prey-independent growth on organic nutrients and predatory growth on live prey. Despite the prey-independent growth, the homolog of *bd0108* did not have typical prey-independent-type mutations. The dual growth mode may reflect the high carbon content of the river, and gives *B. bacteriovorus* Tiberius extended non-predatory contact with the other bacteria present. The HD100 and Tiberius genomes were extensively syntenic despite their different cultured-terrestrial/freshly-isolated aquatic histories; but there were significant differences in gene content indicative of genomic flux and LGT. Gene content comparisons support previously published *in silico* predictions for LGT in strain HD100 with substantial conservation of genes predicted to have ancient LGT origins but little conservation of AT-rich genes predicted to be recently acquired.

**Conclusions:**

The natural niche and dual predatory, and prey-independent growth of the *B. bacteriovorus* Tiberius strain afforded it extensive non-predatory contact with other marine and freshwater bacteria from which LGT is evident in its genome. Thus despite their arsenal of DNA-lytic enzymes; *Bdellovibrio* are not always predatory in natural niches and their genomes are shaped by acquiring whole genes from other bacteria.

## Background

*Bdellovibrio bacteriovorus* (including strains HD100 and 109J) is an intra-periplasmic predator of diverse Gram-negative bacteria. In “attack phase”, the small, vibroid and uniflagellate *B. bacteriovorus* cells collide with and invade bacterial prey wherein they replicate. Despite this provision of nutrition by prey, *B. bacteriovorus* retains a large 3.8 Mb genome, as the sequence of the terrestrial type-strain HD100 [1,2] shows. The gene content is large for two main reasons:

Firstly, entry into and digestion of another bacterium requires coding of an arsenal of hydrolytic enzymes and sensor regulator systems. The evolutionary pay-off is the opportunity to exclusively use the internal contents of another bacterium as food, without competition from other bacteria; the outer membrane of the prey is resealed and macromolecules do not leak from it. In addition, the invaded prey cell, known as the “bdelloplast”, forms a protective barrier against potential environmental insults while the *Bdellovibrio* replicate.

Secondly, lifestyle switching by *Bdellovibrio* to grow prey-independently on conventional carbon sources may also contribute to the large genome size, but this switching has been found rarely in cultivated “attack phase” *Bdellovibrio*. A small fraction of cells (1 in ~10^7^) from predatory laboratory cultures of *B. bacteriovorus* HD100 can be isolated and shown to grow slowly prey-independently on complex peptone-yeast extract based media [3]. These (so-called host-independent, HI) cells grow as long filaments which septate into multiple progeny, some of which are of attack phase size and some are longer serpentine cells. This suggests that some genes for cell elongation, regulatory and alternative degradative-enzymes may be retained in the HD100 genome to allow this alternative, non-predatory lifestyle. The low frequency of isolation host-independently growing *B. bacteriovorus* HD100 or 109J suggests a mutational event is required to activate such prey-independent growth. The site of such mutations has been reported to be in gene *bd0108* which has homology to part of a TypeIVb pilus operon [3,4]. However, it has been impossible to measure the extent to which prey-independent growth of *Bdellovibrio* happens in natural environments, or whether it is a laboratory phenomenon. Thus *Bdellovibrio* are seen as “versitalists” because they prey on diverse bacteria and, at least in the lab, have some prey-independent growth potential after mutational events [3].

Predatory life brings with it an unusual degree of interaction between *Bdellovibrio* and other bacteria, and thus potential sources of lateral gene transfer (LGT). However, the degradation of the prey also includes the degradation from complex polymers (such as in prey genomes and ribosomes) and re-use of prey nucleic acids. Thus a range of nucleases are employed by *Bdellovibrio* and possibly act against LGT during predator–prey interactions [1]. Initial observations, based on a simple examination of GC skew of genes in the *B. bacteriovorus* HD100 genome, suggested that horizontal transfer of foreign DNA was not readily occurring in this bacterium. However, later *in silico* studies of nearest *Bdellovibrio* gene homologues by the Doolittle lab found evidence of ancient LGT from outside the delta-proteobacteria clade [5]. More recently Pan and co-workers used a codon bias analysis of the *B. bacteriovorous* HD100 genome to suggest that AT-rich genes were recently transferred from other bacterial species to *Bdellovibrio*[6]. To date no other *B. bacteriovorus* genome sequences have been published, although Wurtzel and co-workers have described the partial sequence of *B. bacteriovorus* 109J [4]. Therefore in this study, we have analysed the genome of a different strain of *B. bacteriovorus*, which has been freshly isolated from an organically polluted and diversely-bacterially populated river - the River Tiber in Rome [7]. The River Tiber is geographically distant from the german terrestrial site of *B. bacteriovorus* HD100 isolation [2] and has high historic inputs of faecal bacteria, dissolved carbon from sewage, industrial chemicals, plus aquatic and marine bacteria [8]. In this river environment the opportunity for LGT between bacterial species should have been quite high.

Here we provide the first full comparative genomic analysis for *Bdellovibrio bacteriovorus*. We discover that, unlike the lab strain HD100, *B. bacteriovorus* Tiberius can simultaneously grow predatorily and prey/host-independently, extending its previous “versitalist” designation [9] to new extremes. We discuss the impacts on LGT that this has had and provide experimental evidence that concurs with previously published *in silico* evidence from HD100 genome analysis, for the extent of ancient and more recent LGT in shaping the *Bdellovibrio bacteriovorus* genome.

## Results and Discussion

### In contrast to other *Bdellovibrio, B. bacteriovorus* Tiberius grows both prey-independently and predatorily simultaneously

**Figure 1:**
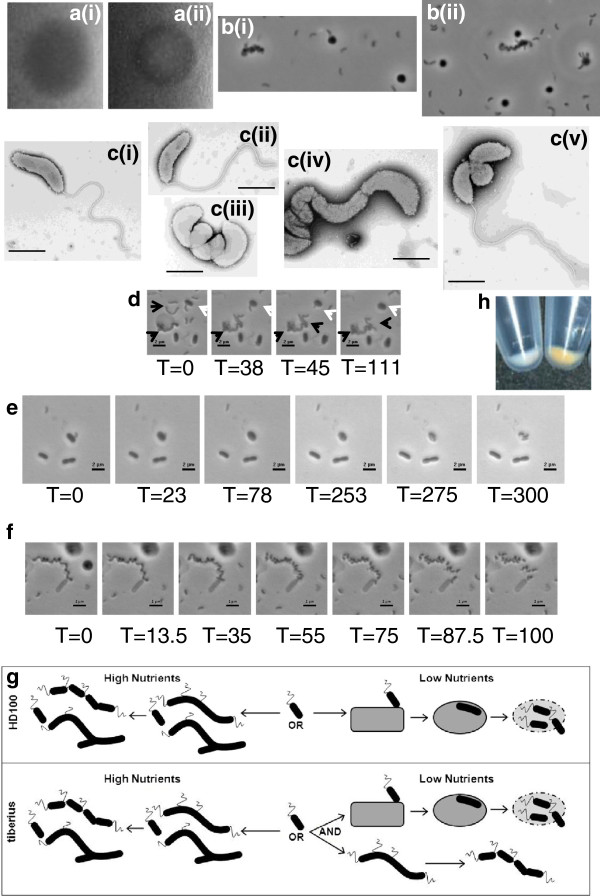
**Simultaneous predatory and prey-independent growth by *B. bacteriovorus *Tiberius.** (**a**) Plaques formed by lysis of prey within lawns of prey cells by *B. bacteriovorus***(i)** HD100 clear plaque and **(ii)** Tiberius plaque with central colony growth. (**b**) Light microscopy images of serpentine prey-independently growing Tiberius cells alongside free-swimming *Bdellovibrio* cells and bdelloplasts. **(c)** Electron microscopy of Tiberius cells (**i & ii**) attack phase, predatory cells (**iii-v**) filamentous, prey-independently growing cells from the same samples as in (i & ii). (**d – f**) Timelapse microscopy still images (from all T= timepoint in minutes from addition of bdellovibrios to slide) from movies showing: (**d**) co-existence of long HI prey-independently growing cells (black arrows) and comma-shaped predatory *B. bacteriovorus* Tiberius invading an *E. coli* prey cell (white arrow); (**e**) evidence that the outcome of prey entry by *B. bacteriovorus* Tiberius results in bdellovibrio replication- one cell enters at T=0 and three leave upon prey lysis at T=300; (**f**) septation by binary fission of the long prey-independently growing form of *B. bacteriovorus* Tiberius. (**g**) Diagram comparing the modes of growth of *B. bacteriovorus* HD100 and Tiberius in both high and low nutrient conditions, showing simultaneous predatory and prey-independent growth by Tiberius in low nutrient conditions. (**h**) Cell pellets of predatory cells showing the white cells of *B. bacteriovorus* Tiberius against the yellow, carotenoid-producing, cells of strain HD100.

Predatory cultures of well-studied *B. bacteriovorus* HD100 and 109J form clear plaques on prey lawns (Figure 1a (i)) and are composed solely of vibroid attack-phase cells. However, pure, predatory cultures of the Tiberius strain formed plaques on *E. coli* lawns each containing a central zone where a small *Bdellovibrio* micro-colony was seen (Figure 1a (ii)). *B. bacteriovorus* Tiberius from the plaques and the predatory liquid cultures derived from them, contained a mix of vibroid and long serpentine morphotypes the latter with spherical regions (Figure 1b and 1c). The long cells resembled axenically-growing prey/host-independent (HI) *Bdellovibrio*, reported by Friedberg [10] and Reiner and Shilo [11]. The distinctive colony-centred plaques continued even after serial passage of the strain, derived from single plaques, predatorily, repeatedly on prey showing that pure cultures contain both cell types. That a pure strain had been isolated was backed up by examination of restriction digest and Southern blot hybridisation patterns for DNA derived from multiple cultures of the Tiberius strain (data not shown) no variation was detected which would have indicated two strains rather than one. The 16SrRNA gene amplified from the genomic DNA of the Tiberius strain cultures gave a single uniform sequence which was used to position the bacterium on a phylogenetic tree (Additional file 1) where it clustered with *Bdellovibrio bacteriovorus* (rather than *Bacteriovorax*) strains, including the type strain HD100 and the well-characterised lab strain 109J [12,13].

Despite the presence of large serpentine cells in cultures; the *B. bacteriovorus* Tiberius were also clearly preying upon, and reducing, *E. coli* prey numbers. Time lapse microscopy (Figure 1d, 1e, 1f) showed simultaneous growth and division of the long serpentine *B. bacteriovorus* Tiberius cells in predatory cultures (suspended in Ca/HEPES buffer, but also containing prey cell debris as a source of amino acids) at the same time as *E. coli* prey in the cultures were being invaded by the small vibroid *B. bacteriovorus* Tiberius cells. The unusual predatory & prey-independent growth behavior of this *Bdellovibrio* was confirmed in three further experimental approaches: These included: (Additional file 2), timelapse microscopy of septating *Bdellovibrio* within a maturing bdelloplast while a prey-independent cell elongated adjacently; (Additional file 3) increasing cell number of a spontaneous streptomycin resistant derivative of *B. bacteriovorus* Tiberius, taken directly from a predatory culture into PY medium, (normally only a tiny percentage - 1 in ~1 × 10^7^- of a predatory *Bdellovibrio* population will grow on PY media); (Additional file 4), evidence by viable counts that *E.* coli were killed and that, concomitantly, *B. bacteriovorus* Tiberius cells increased in number. Thus the processes of predation and HI growth which are separate in previously studied *B. bacteriovorus* strains such as HD100, were occurring simultaneously in *B. bacteriovorus* Tiberius, even when the *B. bacteriovorus* Tiberius was in buffer with prey cells, rather than suspended in a medium rich in organic nutrients, as is required for HI growth by typical HI strains of *Bdellovibrio* (Figure 1g).

The coexistence of prey-independent and predatory cells raises the possibility of cannibalism, with predatory *Bdellovibrio* possibly attacking prey-independent *Bdellovibrio*. This could have explained the sphaeroplast structures observed in many HI cultures [14], so to investigate this possibility, we tagged the abundant nuclear binding protein HuaA with both mCherry and TFP and co-incubated the resulting Tiberius and HD100 strains for several days, both as predatory cultures, HI cultures, and mixtures of the two. Despite this, we saw no convincing evidence of co-incidence of mCherry and TFP within any structures. We therefore conclude that the sphaeroplast structures observed are a normal feature of HI morphology, possibly sometimes caused by damage and not (or at least undetectably rarely) the result of cannibalism. That neither HD100 nor Tiberius strains used each other as prey, is consistent with previous observations for other *Bdellovibrio bacteriovorus* strains. This suggests that *Bdellovibrio* have outer-membranes different to those from the broad range of other Gram- negative outer membranes which they attach to, and invade [15].

### Tiberius and HD100 genomes are highly conserved despite different growth cycle switching controls, but diversity reflective of LGT and different selective pressures in diverse habitats was detected

We had initially chosen to compare the genome of a *Bdellovibrio bacteriovorus* from an environment with many sources of potential LGT, to that of the previously sequenced HD100 strain, to see if the *in silico* predictions of LGT in the HD100 genome were in evidence. Our hypothesis was that predicted “ancient LGT genes” would be more conserved between the two strains and that “recent LGT genes” may differ more between strains. As we discovered experimentally that the Tiberius strain also had differences to HD100 in its predatory versus prey-independent growth control, we also noted genes associated with those phenotypes (such as pilus genes, genes associated with predation, and genes at the *hit* locus including the *bd0108* equivalent).

**Figure 2:**
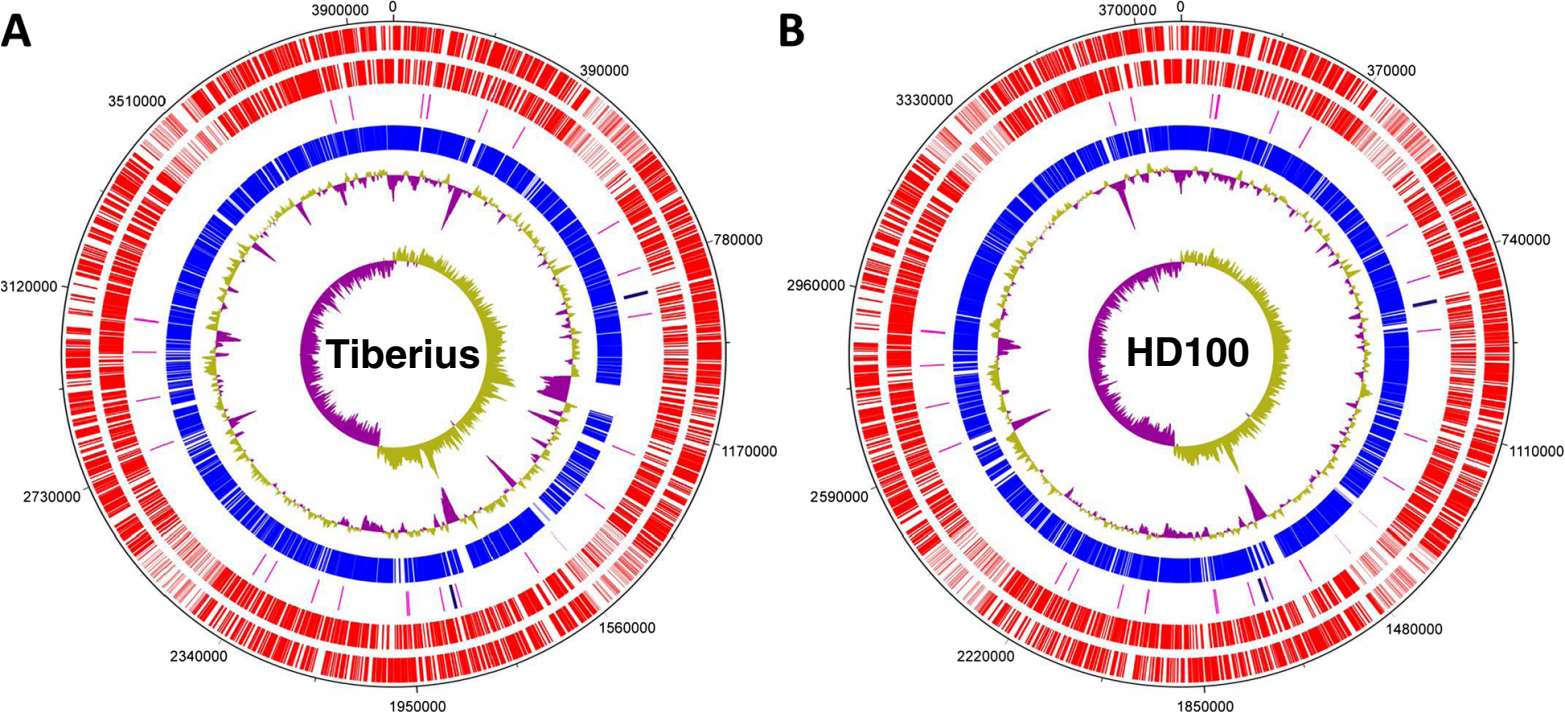
**Whole genome comparison of the *B. bacteriovorus *Tiberius and HD100 strains.** DNAplotter [16] was used to compare the genomes of the (**A**) Tiberius and (**B**) HD100 strains. The circles represent (from outer to inner): CDS on plus strand; CDS on minus strand; tRNAs (pink) and rRNA operons (navy); regions of homology between the two genomes (blue); % GC plot (yellow=above average, purple=below average); and GC skew ([G-C]/[G+C]).

Despite the differences in predatory versus prey-independent lifestyle regulation and terrestrial versus aquatic environments; there was extensive synteny (Figure 2 and Additional file 5), approximate conservation of genome size (3,988,594 bp, 3,738 protein coding genes for *B. bacteriovorus* Tiberius versus 3,782,950 bp, 3,587 protein coding genes for *B. bacteriovorus* HD100), codon usage and conservation of the number and loci of rRNA and tRNA genes (bar one tRNA gene) (Figure 2). There were however differences in genomic content which were analysed further.

Manual BlastP analysis ( http://blast.ncbi.nlm.nih.gov/) was used initially to compare the gene families of each of the predicted proteins encoded by each genome, this would identify genes as “conserved” if they belonged to the same gene families or were paralogous genes. A cut-off of an E-value of 10^-5^ was used as described in materials and methods section. This manual analysis predicted that there were 312 genes present in the Tiberius genome that were not present in the HD100 genome and that there were 256 genes in the HD100 genome that were not present in the Tiberius genome (Additional file 6). Manual BLAST analysis might have been underestimating the number of differences between strains by calling paralogs as if they were orthologs.

Reciprocal Smallest Distance analysis (RSD) [17] was used to identify true orthologs in both genomes as this identified paralogs miscalled manually as orthologs. RSD predicted that there were 3,203 orthologous gene pairs present in both genomes. (Additional files 6 and 7) This inferred that there were 535 genes present in Tiberius but not in HD100 and 384 genes present in HD100 but not in Tiberius (Additional file 6). The sets of unique genes identified by RSD included all 312 genes identified by manual BLAST as unique in the Tiberius strain and all except 2 (*bd1676* and *bd1743)* of the 256 genes identified by manual BLAST as unique in strain HD100. These different genes were either not inherited from the last common ancestor of Tiberius and HD100 and so could represent paralogs on a divergent evolutionary path or examples of LGT, or have been subjected to differential gene loss since the last common ancestor, although this is less likely when the small evolutionary distance between the two strains is considered.

The loci of the genes present only in the Tiberius genome or the present only in the HD100 (Additional file 6) were generally evenly positioned around the *Bdellovibrio* genomes, without strong positional bias for proximity or distance from origin or termination regions with some evidence of gene clusters as well as single insertion-deletions. In the cases of large gene insertions, the differences were obvious on the DNAPlotter Map of each genome by the difference in G+C %, with most areas of insertion corresponding to areas with below average G+C content (Figure 2).

### Mobile DNA elements, translocated genes and gene islands at tRNAs

The Tiberius strain contained more phage and IS element DNAs than the HD100 strain and, suggestive of mobile DNA actions, these may well have contributed to some of the extra gene content in Tiberius versus HD100 although there were no obvious complete phage sequences (Additional files 8 and 9 abc).

TRNAscan [18] showed the presence of 35 tRNAs (Additional file 10) in Tiberius; one less (trnaPro equivalent to HD100 trna0017) than in the HD100 genome. Examination of the codon usage of Tiberius versus HD100 for Pro codons did not show a significant difference, suggesting that “wobble” base pairing by other tRNAs allows translation of Pro codons. The Ribosomal RNA database ( http://rrndb.mmg.msu.edu/index.php) shows that this number of tRNAs is comparably low for a (potentially) free-living bacterium, as those with similar numbers are typically parasites or symbionts. The number of rRNA genes was conserved between strains and they occurred in two sets whose genomic position appeared conserved between strains.

There was a strong locus-association of strain-specific genomic differences with tRNAs, with unique gene islands being found at several tRNAs (Additional file 8), but also at other loci (Figure 2). There were also 4 single different genes with hypothetical annotations inserted uniquely at the following tRNAs: bdt0771, bdt0885, bdt1717 and bdt1771 in strain Tiberius and at tRNA001, tRNA0012, tRNA0013 and tRNA0022 in strain HD100. The prevalence of gene insertion at tRNAs suggests that integrative and conjugative ICE-like elements, which have significant preferences for insertion at tRNA loci [19], could be shaping the evolution of genome diversity in *B. bacteriovorus* by LGT. There were 6 insertions into the Tiberius genome at genes *bdt1194, 1273, 1445, 2597, 2600, 3213* (Additional file 9a) of an identical transposase gene. This encoded a transposase that was homologous (Additional file 9b,c) to those from integrative conjugative elements (ICE) which are associated with genome evolution in enteric pathogens [19]. A region of diversity between strains in the Tiberius genome around *bdt2597-bdt2600* had to be closed by manual sequencing due to assembly of the 454 sequencing being poor due to the two proximal identical ICE elements.

### *In silico* predictions of ancient and modern lateral gene transfer in HD100 are supported by genomic comparisons between HD100 and Tiberius strains

Gophna and coworkers identified 702 genes in the *B. bacteriovorus* HD100 genome that, although their codon usage was not aberrant, had highest homology with genes from other bacterial genera, outside the deltaproteobacteria. They highlighted strongest homology for 421 of these and starred 60 in the SOM to their paper as having potential relevance to predatory activity [5]. They suggested that the lateral transfer of these genes into the *B. bacteriovorus* HD100 genome may have been anciently associated with its predatory evolution. In support of this suggestion we observe (by manual BLAST analysis/RSD analysis) that only 52/57 (7%/8%) of these 702 “anciently laterally transferred genes” found in the HD100 genome were not also present in the Tiberius genome. Of the subset of 60 “ancient LGT” genes which Gophna starred in the HD100 genome as having predicted “predatory relevance”, only 4 (7%) were found by BLAST (*bd1724, 2297,2671* and *3695*) and by RSD analysis these 4 and a further 2 (*bd940* and *1872*) (10%) were found not to be conserved in Tiberius. The finding of ~91-92% of such “anciently acquired” HD100 genes present also in the Tiberius genome suggests, as the Doolittle lab predicted, that they may contribute to shaping the evolution for predation of *Bdellovibrio* and thus that genome flux has not led many of them to be lost, because they employ useful roles and thus their codon usage has become ameliorated to that of the *Bdellovibrio* core codon usage.

In contrast, a more recent study of codon bias in the *B. bacteriovorus* HD100 genome by Pan and co-workers predicted that 35 genes with AT rich codon bias, had been potentially recently laterally transferred from other bacteria [6]. Of these 35, we found (by manual BLAST analysis) that only 5 (14%) were present in the *B. bacteriovorus* Tiberius genome, and this was refined by RSD analysis (rejecting two further paralogs) to only 3 genes (9%) of the Pan and co-workers “recent LGT” list being conserved in both Tiberius and HD100 strains. These genes were *bd0008, bd0246 and bd3708*. This finding lends support to the idea that these 35 genes may have been laterally acquired more recently in evolution in *B. bacteriovorus* HD100 and thus are not necessarily found in the Tiberius strain.

Thus the experimental isolation and sequencing of the Tiberius strain, from a different environment to strain HD100, does support the *in silico* predictions [5,6] garnered from analyses of phylogeny and codon usage in the genome of the single type strain HD100 by evolutionary geneticists.

### Potential significance and LGT sources of genes present in *B. bacteriovorus* Tiberius, but not HD100

The Tiberius strain genome contains 312/535 genes that manual Blast/RSD analysis (Additional file 6) defines as not present in the genome of HD100. Approximately half of these are designated hypothetical and 10 -15% conserved-hypothetical. Some of these gene products (Bdt1104- haloacid dehalogenase, Bdt1074, Bdt2739 and Bdt3264 beta-lactamases, Bdt2421 nitrilase, Bdt2998 intradiol dioxygenase, Bdt2208 urea decarboxylase associated protein) may be associated with the degradation, by prey-independently growing *B. bacteriovorus* Tiberius, of molecules from a river that is reported to be polluted with aromatic hydrocarbons, industrial and farming effluents and sewage [7,8]. Other Tiberius-only genes (*bdt0608, bdt1429, bdt1438, bdt1888, bdt2734, bdt2783, bdt3127*) are predicted to encode transporters that may efflux toxic compounds found in the river.

More than twenty Tiberius-only genes are suggestive of mobile DNA transfer events including the 6 identical IS element genes mentioned above and the additional phage/nuclease genes (*bdt0236, bdt1197, bdt1452, bdt1735, bdt2336* and *bdt2713-4*), nucleic acid modifying genes and toxin antitoxin genes (*bdt1697-8*). Of the Tiberius-only genes from the manually examined BLAST analysis, there were 10 putative regulatory genes whose products may have roles in the simultaneous predatory and prey-independent growth of Tiberius: two with sigma factor homology and 5 with MarR, Fur, LysR, AraC or other regulator homologies. 50 genes were predicted to encode proteins with modifying (methylation; glucosyl or glutathione transferase) or hydrolytic activities against proteins and carbohydrates, including peptidoglycan deacetylase and lytic transglycosylase activities which may facilitate new pathways of cell wall growth.

15 Tiberius-only genes had predicted products that may modify the *Bdellovibrio* cell surface including putative adhesions, LPS modification or pilus fibre modification genes and secreted and membrane proteins, including a single PAS domain putative sensory protein. These gene products would reflect different surfaces present in the river environment for adhesion. There was a single gene, *bdt3408*, with *relA/spoT* homology that may encode the alteration of ppGpp levels- this is worthy of future investigation as ppGpp signals bacterial amino-acid starvation and the abnormal co-growth of both prey-independent and predatory *B. bacteriovorus* Tiberius in dilute buffers plus prey cell debris only, could be affected by this.

LGT will account for the presence of some of these Tiberius specific genes and evidence in support of this came from looking at the microbial source of the highest similarity homologue. These included aquatic and marine bacteria: Cyanothece (3 genes), *Acaryochloris* (3 genes)*, Anabaena* (2 genes)*, Nostoc*(1 gene)*, Roseiflexus* (2 genes)*, Rhodospirillum* (2 genes)*, Lutiella* (1 gene)*, Hahella* (3 genes), *Marinomonas* (2 genes)*, Microscilla* (2 genes), *Marinobacter* (1 gene), and *Natronamonas*, (1 gene), an archaeon from high salt, ammonia rich environments. This totals 23 of the 143 tiberius-only genes (as found by our Blast analysis) with homology to genes from members of other bacterial species*.*

There were a few examples of evidence of transfer from soil and plant associated bacteria into Tiberius, including *Rhizobium* and *Pseudomonas* (9 of 143)*.* There was evidence suggestive of transfer (by highest BLAST scores) occurring with genes from some pollutant bacteria, reported to be in the Tiber such as *Clostridium* and others including *Vibrio*, *Eggerthella* and *Shewanella* species (totalling 8 of 143), which would be common in a faecal pollutants or anaerobic sludges [20]. These include bacteria that *Bdellovibrio* do prey upon, but did not include *Salmonella* and *E. coli* which are known to colonise the Tiber [7,8,20] and which are commonly used *Bdellovibrio* prey in the lab.

The Tiberius-only gene set will also include some ancestral genes, shared with a common deltaproteobacterial ancestor, which have been lost by genome flux from the HD100 genome but retained as they confer fitness to Tiberius. Evidence suggestive of these was seen by highest scoring BLAST homologues being from the deltaproteobacteria, including the Myxobacteria such as *Myxococcus, Stigmatella* and others and the metal reducing deltaproteobacteria including *Geobacter* and *Desulfovibrio*. Some of the genes in this category may encode products relevant to prey-independent growth in the polluted environment of the Tiber [8]. They include *bdt3058*, a polyhydroxybuturate depolymerase gene whose highest homologue is from *Myxococcus*- this might allow degradation of recyclable plastic pollutants. In addition the gene *bdt3408* with relA/spoT homology mentioned above has highest homology with a gene from *Geobacter* and a *tetR* transcriptional regulator gene has highest homology with *Desulfuromonas*, both deltaproteobacteria.

### Potential significance and LGT sources of genes present in HD100 but not in Tiberius

Again these genes (256/384 by manual BLAST/RSD analysis) will fall into categories of ancestral genes lost by Tiberius or genes laterally transferred into HD100. Genes present in HD100 but absent from Tiberius include two associated with cyclic-diGMP signalling function- a GGDEF diguanylate cyclase-synthase gene *bd3766* and an HD-GYP-family gene, *bd3880*, predicted to encode c-diGMP degrading or binding function; however 4 other GGDEF genes and 5 other HD-GYP genes were also in the Tiberius genome, allowing for c-diGMP signalling; furthermore, studies on the *bd3766* GGDEF gene product in HD100 showed that unlike the other four GGDEF genes, *bd3766* does not produce a strong growth phenotype when deleted from strain HD100 [21].

A large group of genes running from *bd1677-bd1702*, predicted to be associated with outer membrane and capsular polysaccharide synthesis, were present in strain HD100 only. These differences likely reflect different habitats of HD100 possibly binding to other micro or macro-organism surfaces in soil. These interactions would be are absent in the aquatic niche and so the nature of the surface capsule would be different. Another category of genes present in HD100 but absent from Tiberius appear to be associated with encoding alternative electron transport. These include some cytochrome biogenesis genes, including *bd2046, bd2668* and, *bd2597-bd2602,* a small putative nitric oxide reductase gene cluster. This difference is suggestive of alternative electron transport to nitrogen compounds probably relevant to the nitrogen cycles of the HD100 terrestrial and rhizosphere niches.

Four HD100 genes *bd1723-bd1725* and *bd1730* associated with carotenoid synthesis are not conserved in Tiberius, suggesting that free radical protection in terrestrial and aquatic environments is achieved by different pathways. This carotenoid gene content seems to correlate with the appearance of *B. bacteriovorus* Tiberius cells which are white, compared to the yellow colour of the HD100 strain (Figure 1h). The absence in Tiberius of the HD100, *kdpA-E* genes *bd1755-bd1759* may fit with different “compatible solute” adaptation strategies to changes in osmolarity between aquatic and terrestrial strains.

More typical examples of “mobile DNA”, present in HD100 but not Tiberius include phage genes and also *bd3694-bd3696* genes for a restriction-modification system. In contrast to Tiberius where a diverse range of bacteria were potential sources of LGT, the most common BLAST top hits for products of genes present in HD100 but not in Tiberius were other deltaproteobacteria which are terrestrial, such as *Myxococcus*, *Geobacter* and *Stigmatella*, followed predominantly by other terrestrial bacteria such as *Hyphomicrobium* and plant associated bacteria such as *Pseudomonas syringae* and *Azocarus sp,* rarely there were bacteria of a less certain habitat, including: *Flavobacterium* and *Serratia*. The HD100-only genes with deltaproteobacteria BLAST top hits may represent ancestral genes, or LGT from soil bacteria.

### Rates of synonymous, non-synonymous substitutions in orthologous gene pairs

The RSD analysis allowed us, for 3203 orthologous pairs of genes in the two strains, to compare the rates of synonymous and non-synonymous substitutions, and omega the ratio of dN/dS (Additional file 7). While population genetics theory predicts that genes with dN/dS > 1 would be under positive selection, in reality positive selection is likely to be acting only on a limited number of sites in a gene. Thus some gene pairs with high dN/dS (omega) values (but not >1) are discussed in this section.

Consistent with findings for other bacterial species [22] the pairwise comparison of orthologs from *Bdellovibrio* HD100 and Tiberius suggest that these genomes are evolving under strong purifying selection (median dN/dS = 0.041). Exceptions to this include a number of genes with high omega values, which may be experiencing positive selection due to adaptive evolution or relaxed purifying selection due to differences in functional requirements arising from the different niches. Gene pairs discussed below with high omega values (> 0.1) also have dN > 0.01 and dS < 1.5, (Additional file 7), which allowed meaningful estimates of omega.

Genes with high rates of non-synonymous substitutions may be: 1) Ancestral genes that are subject to evolutionary pressures in one strain, because the encoded products confer a different fitness in *Bdellovibrio* in the terrestrial niche versus the aquatic one. An example of this category is the *pilA* gene (*bdt1269/bd1290)*; the product of which (a pilus fibre) is subject to external environmental selective pressures (phage and protozoal attachment sites for example, or different prey surfaces encountered in different niches) and so is typical of a gene that would be fast evolving in the different aquatic versus terrestrial environments of the two *Bdellovibrio* strains. 2) Genes acquired by LGT, which are subject to ongoing amelioration. The *cinA* homologue *bdt0498/bd0510* gene pair are an example of this as the gene lies immediately upstream of *recA* in each genome but has different codon usage from the surrounding 5’ and 3’ regions. 3) Paralogous genes where there has been a duplication event such that one paralog is able to be changed by random mutagenesis to encode a paralogous rather than orthologous function in a new niche. An example of this latter category is the *bd2572/bdt2498 tatA* gene pair, which encode a structural component of the folded-protein secretion- apparatus called the TAT system. Gene *tatA* has a paralog *tatE (bd2196* in HD100), [23] likely allowing more rapid sequence divergence of *tatA* as evidenced by the high omega score.

### A comparison of the HD100 strain Hit locus gene *bd0108* to Tiberius gene *bdt0101* in light of the Tiberius prey-independent growth phenotype

When *B. bacteriovorus* HD100 or other strains convert to prey-independent growth in the lab, often, (but not always), a concomitant mutation in gene *bd0108* can be detected [24]. Such mutations often abolish expression of the *bd0108* gene product (which is 101 aa), or commonly involve the deletion of a 42 bp region flanked by repeat elements. The Tiberius genome contains a same-length homologue of *bd0108*, gene *bdt0101.* Although there are 3 amino-acid differences between the predicted gene products: V^HD100^31A^tiberius^, A86T and the less conservative substitution G97S; none of these substitutions have been reported in other *Bdellovibrio* to cause prey-independent growth. Thus, although we do not have absolute proof; *bd0108* substitutions are not likely to be the cause of the simultaneous predatory and prey-independent growth observed in the Tiberius strain. As the Tiberius source DNA which was used for the genome sequence contained mostly attack phase cells, with some of the longer prey-independently growing cells; it seems unlikely that mutations in the *bdt0101* gene were required for this dual growth mode. This was confirmed by isolating (by conventional *Bdellovibrio* methods), HI growth phase Tiberius cells. This was done by screening and selection, in the absence of prey cells, on PY medium [14] and re-sequencing the *bdt0101* gene. For 6 separate HI isolates of Tiberius, the *bdt0101* sequence was unchanged from the wild-type. This suggests that HI growth may be regulated by different mechanisms in the Tiberius and HD100 strains. It is a possibility that these differences are associated with the above described SNPs in these genes, but testing this would be the subject of a further study.

### Conservation of encoded functions in the Tiberius genome relevant to prey location in the HD100 strain

Motility and chemotaxis have both been shown to be important in efficient prey location by *B. bacteriovorus* HD100 when analysed in a laboratory setting [25,26]. All HD100 flagellar-related genes are conserved in the Tiberius genome, including the multiple copies of genes encoding the structural flagellin FliC (6 copies), the motor proteins MotA and MotB (3 each) and the rotor protein FliG (2 copies). The two key flagellin genes for motility, *bd0604* encoding FliC3 (the founder flagellin without which no filament is assembled in HD100) and *bd3052* encoding FliC5 (a major flagellin, without which cells with short flagella and slow motility are made) are predicted to encode products 100% identical at the amino-acid level in HD100 and Tiberius.

The chemotaxis system “directs” flagellar motility to prey-rich regions, and all tactic signalling proteins, including the multiple copies of key phospho-relay signalling components (CheY 5, CheW 3, CheA 3) are conserved. The number and diversity of the MCP tactic sensor genes differs between the two strains. 14 MCP genes and the single aerotaxis gene are conserved in both strains. A pair of MCP genes from HD100 (*bd1872* and *bd1873*) are fused into a single gene in Tiberius (*bdt1841*). The HD100 genome contains an additional 4 MCP genes (*bd0262*, *bd0932*, *bd2596* and *bd2622*) that are not found in Tiberius, whilst the Tiberius genome also contains a further 4 MCP genes not found in HD100 (*bdt1069*, *bdt1102*, *bdt1169*, *bdt1593*). The different aquatic and terrestrial environments from which the two *Bdellovibrio* strains have been isolated may have required chemotactic responses to different input stimuli, resulting in alterations to genes encoding the MCP-stimulus sensor complement of chemotactic sensory array components.

Type IV pili have been shown to be essential for predation by *B. bacteriovorous* HD100 [27] and in strains 109J and *B. exovorus* JSS [28]. All genes predicted to be involved in the synthesis of Type IV pili in HD100 (as identified in [27] and [29]) were conserved in the Tiberius genome. Both *B. bacteriovorus* HD100 and Tiberius have an incomplete (and matching) set of flp-pilus-associated genes, yet neither have a significantly convincing homologue for the structural flp fibre subunit. The HD100 genome contains none of the genes required for fimbrial production, whilst the Tiberius genome contains inserted genes coding for the chaperone Bdt0200 PapD and Bdt0201 – PapC; but again no homologue to encode the structural fibre subunit FimA. It may be that novel fimbrial fibre subunit proteins are encoded in these genomes but bear no resemblance to those previously studied in other bacteria. Four operons encoding Tol-Pal-TonB-like proteins for gliding motility are conserved in both Tiberius and HD100 strains and the Tiberius strain was observed to glide on agarose surfaces as does HD100 (data not shown, [30]).

### Conservation of genes in Tiberius whose transcription in HD100 is associated with predatory or prey-independent *Bdellovibrio* lifestyles

Previously we profiled by microarrays the proportion of the HD100 genome that was expressed in conditions of early predatory invasion of prey, versus prey-independent growth or non-replicative life in buffer seeking prey (“attack phase”) [31]. This revealed, for strain HD100, subsets of genes predicted to be associated with each lifestyle. Comparing the extent of genome conservation for Tiberius with each HD100 transcriptome shows that of the 256–384 manual BLAST-RSD predicted (in this order of numbers x-y; with x representing BLAST and y representing RSD numbers respectively throughout this section) genes not conserved between Tiberius and HD100:

154–232 (~60%) of the non-conserved genes were found to be associated with “attack phase” life in HD100 (downregulated in HI growth conditions compared to “attack phase” life in buffer in studies by Lambert and co-workers [31]). It is not surprising given the different terrestrial versus polluted aquatic environments from which the two strains were isolated, that the major gene differences lie in genes expressed in HD100 while it was in predatory mode, hunting prey as viability to encounter prey would be enhanced by repelling negative influences in the terrestrial environment. It must be remembered though that for HD100 the majority of the population cannot grow and divide in such a state, without prey entry, unlike the Tiberius strain. Of these 154–232 genes, 21–27 are predicted (by PSORTb) to localize to the inner membrane and 5 (for both BLAST-RSD sets) are predicted to localize to the outer membrane supporting the idea that at least some have functions associated with the differing external environments encountered by the two strains.

Only 16–25 (~6-7%) of the non-conserved genes were found to be transcriptionally profiled in HD100 as “predatory invasion associated” (Found to be upregulated at 30 minutes of prey invasion by HD100 compared to attack phase [31]). This means that 224 by BLAST-215 by RSD, of the 240 predatorily associated genes found in the *B. bacteriovorus* HD100 “predatosome” [31] are conserved between the two different *Bdellovibrio* strains and likely encode products vital to predatory processes.

31–40 (~10-12%) of the non-conserved genes were found to be “HI or growth associated” (Found to be upregulated in HI growth of HD100 compared to attack phase [31]). This shows that 1063–1054 genes (96-97%) of 1094 identified in HD100 transcriptomics as HI growth associated (including, primary metabolic, division and genome replication genes i.e. “housekeeping genes”) are conserved between the two strains.

The remaining 55–87 (~21-23%) of the 256 HD100 genes, not conserved in Tiberius, were not differentially regulated in the HD100 transcriptomic studies to date and may be associated with stages of the lifecycle not transcriptionally profiled in our previous study (i.e. later in the predatory cycle after the 30 minute prey-interaction timepoint that was previously studied [31]).

Thus the genomic comparison of Tiberius and HD100 genomic content does show conservation of predatorily associated genes as would be expected by their conserved prey invasive life styles, albeit in environments where the prey and the physico-chemical environments differ.

## Conclusions

The novel *B. bacteriovorus* Tiberius strain grew simultaneously predatorily and prey-independently which may be related to the highly polluted niche containing, in addition to river and esturine bacteria, industrial effluents, organic carbon and bacteria from human sewage [7,8].

The genome of aquatic *B. bacteriovorus* Tiberius showed significant conservation with that of terrestrial *B. bacteriovorus* HD100, especially in the case of 240 genes profiled by transcriptional studies in strain HD100 (Lambert & co-workers [31]) to be specifically associated with early events during prey invasion. As there have not been extensive genomic sequencing studies of the predatory bdellovibrios, this conserved subset of 224 (by BLAST) 215 (by RSD) “predatosome” genes illustrate which is required for predatory invasion and may be worthy of further mutagenesis and study. Only one of these genes, *bd0697,* predicted to encode an actin-binding protein, is also predicted by Gophna and Doolittle to have been acquired by ancient lateral gene transfer [5]. It is possible that this gene could encode binding of *Bdellovibrio* to prey MreB (actin homologue) cytoskeletons for predatory invasion. However this speculation requires experimental verification. It is likely that amelioration & co-evolution of the *Bdellovibrio* genome has masked the identity of some anciently acquired predatory genes.

The diverse bacterial microbiota of the Tiber afforded the *B. bacteriovorus* Tiberius strain many chances for predation, but also for LGT from bacteria that it may not have preyed upon. The differences and similarities in gene content between the two *Bdellovibrio* strains, map well onto the conclusions of Pan et al. and Gophna and Doolittle who made *in silico* predictions of, respectively, modern and ancient LGT into the *B. bacteriovorus* HD100 genome. Only 8% of genes predicted to have a modern LGT source in *B. bacteriovorus* HD100 [6] were conserved in *B. bacteriovorus* Tiberius, but far more, 92%, were conserved in both strains from the group predicted to be as a result of ancient LGT into strain HD100 [5].

When considering possible LGT in each strain it was found that genes present in strain HD100, but not Tiberius, commonly encoded products with BLAST top-hits from terrestrial or plant-associated bacteria. In contrast those genes found in Tiberius but not HD100, commonly had BLAST top-hits from aquatic and marine bacterial sources, but not from *E. coli* and *Salmonella*, which are experimentally documented by Bonadonna and co-workers [7] to be in the River Tiber, as sewage pollutants, in ranges from 10^1^ - 10^3^ ml^-1^. This is an interesting finding as although *E. coli* and *Salmonella* are readily preyed upon by *B. bacteriovorus* Tiberius, the predation efficiency or even capability on the marine and aquatic bacteria has not been determined, but should be in future environmental microbiology studies. Any differences in predation efficiency could suggest that LGT occurs into *Bdellovibrio* from bacteria which are sub-optimal prey. *Bdellovibrio* invade other bacteria using their non-flagellar “nose” pole only. Non-predatory contacts by other bacteria at points elsewhere on the *Bdellovibrio* cell, do allow conjugation to occur (as is employed experimentally for plasmid transfer in the lab) and thus this could be a source of LGT via plasmids in the wild. The fact that a percentage of *B. bacteriovorus* Tiberius populations spend their lives in prey-independent, rather than predatory growth, unlike the dominantly predatory HD100 strain, might contribute to a greater propensity for LGT into the Tiberius strain (which does have a larger genome). However the diverse dissolved solutes of the Tiber also may place a selective pressure on the *B. bacteriovorus* Tiberius strain to retain more genes encoding efflux pumps and detoxifying enzymes that HD100.

This study shows that there is a balance between predatory and prey-independent growth in some *Bdellovibrio* such as strain Tiberius, from natural environments. Self recognition of *Bdellovibrio* does occur across strains and the HD100 and Tiberius strains did not prey upon each other.

Despite their predatory nature, and efficient degradation of prey bacterial nucleic acids, aquatic and terrestrial *B. bacteriovorus* do acquire DNA from bacteria in the wild. In this study LGT into *B. bacteriovorus* Tiberius came more from natural microbiota of the aquatic-marine niche of the River Tiber than from sewage pollutant bacteria.

## Methods

### *B. bacteriovorus* Tiberius isolation and biological studies

A water sample was collected from the River Tiber in Rome (41°53'59.82"N 12°27'50.19"E) and was mixed in a 50:50 (v:v) ratio to a mixture of Ca/HEPES buffer (2 mM CaCl_2_, 25 mM HEPES pH 7.6) and *Escherichia coli* S17-1 prey (giving an average starting *E. coli* concentration of 1 × 10^8^ per ml); this was incubated at 29°C with shaking at 200 rpm. *E. coli* were used as prey because the River Tiber is reported to have a high *E. coli* content [7]. Invaded prey ‘bdelloplasts’, formed by *Bdellovibrio,* were seen by light microscopy and the enrichments were then serially diluted and plated onto double-layer YPSC agar plates with *E. coli* S17-1 prey and incubated until well separated individual *Bdellovibrio* plaques were observed in prey lawns. Each plaque was added to fresh Ca/HEPES buffer with *E. coli* prey until prey lysis and small *Bdellovibrio*-like cells were observed before the plating was repeated. This was repeated three times (each time selecting a single, well isolated plaque from the dilution plates) giving a culture that contained a single strain of *Bdellovibrio* preying upon the *E. coli* prey (Isolate *Bdellovibrio bacteriovorus* Tiberius isolate B5B). Purity was verified by microscopy, by consistency of SDS PAGE total protein patterns and by 16SrRNA PCR with both general-bacterial and *Bdellovibrio*-specific primers [12].

### Video microscopy

Time-lapse video microscopy was carried out as described previously [32]; 10μl samples of *Bdellovibrio bacteriovorus* or *B. bacteriovorus* mixed with *E. coli* prey bacteria were spotted onto solid 1% agarose pad surfaces in Ca/HEPES (2 mM CaCl_2_, 25 mM HEPES pH7.6) buffer and covered with a cover slip. Microscopic images were acquired at room temperature every 150 seconds over several hours, with a Nikon Eclipse E600 microscope with 100x phase contrast and Simple PCI software (version 5.3.1.081004). The resulting images were then encoded into time-lapse movies at 7 fps.

### Electron microscopy

*B. bacteriovorus* Tiberius morphology was assessed after 24 hours of growth in Ca/HEPES buffer with *E. coli* S17-1 prey under standard conditions. *Bdellovibrio* cells were stained with 1% phosphotungstic acid (PTA) pH 7.0 and imaged with a JEOL 1200EX electron microscope at 80 kV.

### Genome sequencing, assembly, annotation and content analysis

For DNA purification *B. bacteriovorus* Tiberius were pre-grown predatorily with *Pseudomonas putida* as prey. (*P. putida* has a GC-rich genome and was prey in the *B. bacteriovorus* HD100 genomic sequencing project, to contrast with the predator 50% AT:GC content [1]). After 24 hours the predatory culture was checked for prey lysis and passed twice through 0.45 μm filters to remove any residual prey. This step will also exclude many but not all, of the larger, prey-independently-growing *Bdellovibrio* cells as they are thin enough for some to pass through the filter end-on. Genomic DNA was prepared from 2.5 × 10^9^ of these filtered *Bdellovibrio* cells with a Sigma bacterial genomic DNA kit. The resulting genomic DNA was subjected to 454 DNA sequencing by Cogenics, France (now a division of Beckman-Coulter USA). This resulted in 60 fold coverage of 16 contigs which we joined by PCR and further DNA sequencing by Source Bioscience (UK) to form a contiguous sequence file. In most cases solely 1–3 bp or a single phosphodiester bond joined contigs. There was one large gene island at *bdt2602* where further conventional sequencing joined the genome via a fragment containing two copies of the IS2 element transposase *insD* with other surrounding genes. This Tiberius genome locus corresponds to *bd2666-82* in the HD100 genome where a different large gene island was inserted at a tRNA (see results below). Thus we assembled and annotated a single genome of 3,988,594 bp from DNA of the morphologically-divergent aquatic *B. bacteriovorus* Tiberius strain and examined its synteny and differences to the terrestrial *B. bacteriovorus* HD100 lab strain which was isolated in the 1960s and sequenced in 2004 [1].

Nucleotide and amino-acid sequence analyses were carried out with the BLAST programs at NCBI (National Center for Biotechnology Information, USA). An automatic annotation of the genome was carried out by submission to the xBASE bacterial genome annotation site ( http://www.xbase.ac.uk/annotation/[33]), using *B. bacteriovorus* HD100 [1] as a reference genome. The annotation pipeline included the use of Glimmer [34] for gene prediction, tRNAScan-SE [18] for prediction of tRNA genes, RNAmmer [35] for ribosomal RNA prediction, and protein BLAST [36] against the reference genome (*B. bacteriovorus* HD100 [1]) for gene annotation. This annotation was then manually edited using the Artemis and ACT II suite of programmes (Sanger Centre UK) and BLAST analysis [36], as above, was used to identify homologous genes between both strains, those which were not conserved, and those potentially laterally transferred (for the gene products of which a strong BLASTP homologue (e≤−05) was found from a different bacterium).

The completed genome sequence was submitted to Genbank (USA); submission number GDsub15515 and accession number CP002930.

### Rates of evolution by genome wide analysis

To identify orthologs between *B. bacteriovorus* strains HD100 and Tiberius, we used the Reciprocal Smallest Distance (RSD) algorithm [17]. This algorithm combines reciprocal best BLAST hits with estimates of evolutionary distance to identify orthologous pairs of genes. Incorporating evolutionary distance improves detection of orthologs when paralogs are also present, such as the gene duplication events observed in *Bdellovibrio*. Due to the close evolutionary distance between HD100 and Tiberius, we used stringent settings for the e-value cutoff (10^-20^) and the divergence cutoff (0.2).

For each orthologous pair identified by RSD, we used a set of (Duke University) in house scripts to invoke TransAlign.pl [37], which generates an amino acid-based alignment and then back translates to obtain the nucleotide sequences. Each pairwise nucleotide sequence alignment was analyzed in PAML [38] to estimate dN (average number of nonsynonymous changes per nonsynonymous site) and dS (average number of synonymous changes per synonymous site). This allowed us to interrogate the rates of evolution in orthologous gene pairs between strains.

## Abbreviations

LGT: Lateral gene transfer; HI: Host-independently growing Bdellovibrio derived directly by screening and selection for growth on artificial media containing peptone and yeast extract; ICE: Integrated conjugative element; RSD: Reciprocal smallest distance (between two genes); ACT II: Artemis genome comparison tool.

## Competing interests

There are no competing interests.

## Authors' contributions

RES and LH conceived the study, LH, CL and DSM carried out biological studies and microscopy. LH and TRL closed the genome with assistance from RT. LH formatted the genome sequence and led with RES the manual annotation/analysis of LGT which was carried out by TRL, CL, RT, SMB, MJC, DSM, AKF, RJA and MATSH. LW suggested and carried out RSD and PAML analyses of gene evolution with inputs and software from JJW who asked to be acknowledged on the paper. RES wrote the manuscript with co-authoring inputs and figures from LH, CL, LW and editorial comments from the other co-authors. All authors read and approved the final manuscript.

## Supplementary Material

Additional file 1 **Phylogentic tree of the*Bdellovibrionales*including*B. bacteriovorus*Tiberius.** Neighbour-joining tree of the 16S rRNA sequences of the *Bdellovibrionales*. *B. bacteriovorus* Tiberius is shown by its isolate number ‘b5b’. Tree produced using the in-built neighbour-joining algorithms in the Arb program, and bootstrapped using n=500. *Myxococcus xanthus* Mx1622 was used as an outgroup to root the tree. Click here for file

Additional file 2 **Stills from timelapse microscopy video showing simultaneous growth and division of *B. bacteriovorus *Tiberius: A filamentous *B. bacteriovorus *Tiberius grows, divides and fragments in a large *E.coli *prey bdelloplast (lower arrow) while a smaller *B. bacteriovorus *Tiberius (upper arrow) elongates prey-independently.** Shown by time lapse microscopy from a predator-prey culture in calcium-HEPES buffer, supported on an agarose pad on a microscope slide. Click here for file

Additional file 3 **Graph showing *B. bacteriovorus *Tiberius host-independent growth of cells from a predatorily grown culture: Spontaneously streptomycin-resistant Tiberius cells were taken directly from a culture grown on prey cells.** They were then directly grown in PY broth (Horowitz et al., 1974) in the presence of streptomycin to inhibit growth of prey *E. coli* (still present in the co-culture). The resulting Tiberius growth was measured as an increase in OD at 600 nm, shown in the graph against time of incubation at 29°C. The larger prey-independent cells of Tiberius were large enough to measure by optical density (although attack phase sized *Bdellovibrio* are not) , thus the OD600nm values are probably an under-estimate of the growth rate of total *Bdellovibrio* (including smaller cells) in the population. The data shown are from 3 independent repeats and the error bars are 1 standard deviation from the mean. Click here for file

Additional file 4 **Graph showing evidence of killing of *E. coli *S17-1 and growth of *B. bacteriovorus *Tiberius in a predatory culture.** Start- and end-point analyses of viable cell numbers of *E. coli* prey cells and Tiberius predatory cells by plaque (Tiberius) and colony (*E. coli*) counts on soft agar overlays on prey lawns, or conventional LB agar plates, respectively. Click here for file

Additional file 5 **ACT comparison of B. bacteriovorus HD100 and Tiberius genomes.** HD100 is shown along the top of the comparison, with Tiberius below. Areas shown in red are regions of high synteny; blue lines represent areas of homology but in reverse orientation. Click here for file

Additional file 6 **Unique Genes called by RSD and by Manual Blast.** First page shows the annotation of all genes in each of the HD100 and Tiberius genomes alongside whether they were identified as being unique by either RSD or blast analyses. Second page shows only those genes identified as unique by either analysis method, listed by Bd# or Bdt#. The third page again shows the unique genes, this time listed by analysis method. Click here for file

Additional file 7 **Evolutionary rates analysis for all 3203 orthologous genes in *B. bacteriovorus *HD100 and Tiberius predicted by RSD.** Substitution rates were estimated by pairwise comparison in PAML. Red highlighting denotes pairs with dS > 1.5, which indicates saturation of synonymous sites. Table title headings are as follows: ortholog pair (from RSD) = this is a code assigned by our in-house scripts; sites = number of nucleotides (minus the stop codon); dN = average number of nonsynonymous changes per nonsynonymous site; dNse = standard error of dN (calculated by the curvature method); dS = average number of synonymous changes per synonymous site; dSse = standard error of dS (calculated by the curvature method); lnL = likelihood of the evolutionary model used in PAML analysis; t = sequence distance estimated by maximum likelihood; kappa = estimated transition/transversion rate ratio; omega = dN/dS. Click here for file

Additional file 8 Table showing the location and composition of unique gene islands located next to tRNAs. Click here for file

Additional file 9 **a. DNA encoding ICE like transposase element, 6 identical insertions of which were unique to the *B. bacteriovorus *Tiberius genome.b.** predicted domains on encoded protein from Blast X search. **c.** Alignment of Tiberius encoded IS element product translated from bases 7–732 with insertion element IS2 transposase catalytic protein InsD of *E.coli.* The amino-acid sequences are 48% identical. Click here for file

Additional file 10 **Table showing the location and type of each tRNA identified by tRNAScan-SE in the genomes of *B. bacteriovorus *HD100 and Tiberius.** Note the absence of the Pro-type tRNA from the Tiberius genome. Click here for file
