# Supplementary figures and images for: Genome analysis of a simultaneously predatory and prey-independent, novel Bdellovibrio bacteriovorus from the River Tiber, supports in silico predictions of both ancient and recent lateral gene transfer from diverse bacteria
Source: BMC Genomics. 2012 Nov 27;13:670. doi: 10.1186/1471-2164-13-670 (PMC3539863; doi:10.1186/1471-2164-13-670)

## Slide 1
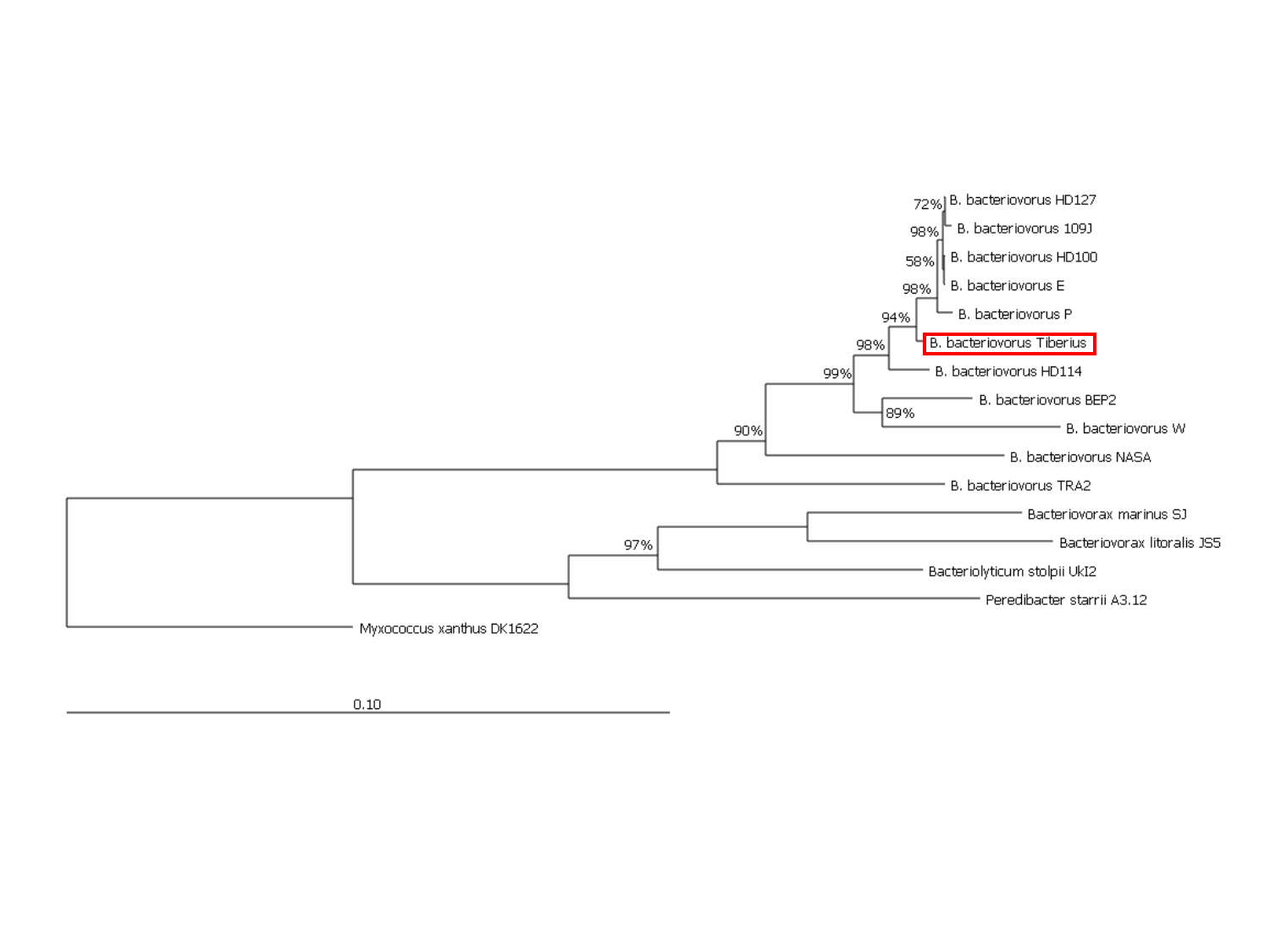

Supplement: Additional file 1 — Phylogentic tree of theBdellovibrionalesincludingB. bacteriovorusTiberius. Neighbour-joining tree of the 16S rRNA sequences of the Bdellovibrionales. B. bacteriovorus Tiberius is shown by its isolate number ‘b5b’. Tree produced using the in-built neighbour-joining algorithms in the Arb program, and bootstrapped using n=500. Myxococcus xanthus Mx1622 was used as an outgroup to root the tree. [file 1471-2164-13-670-S1.ppt]

## Slide 1
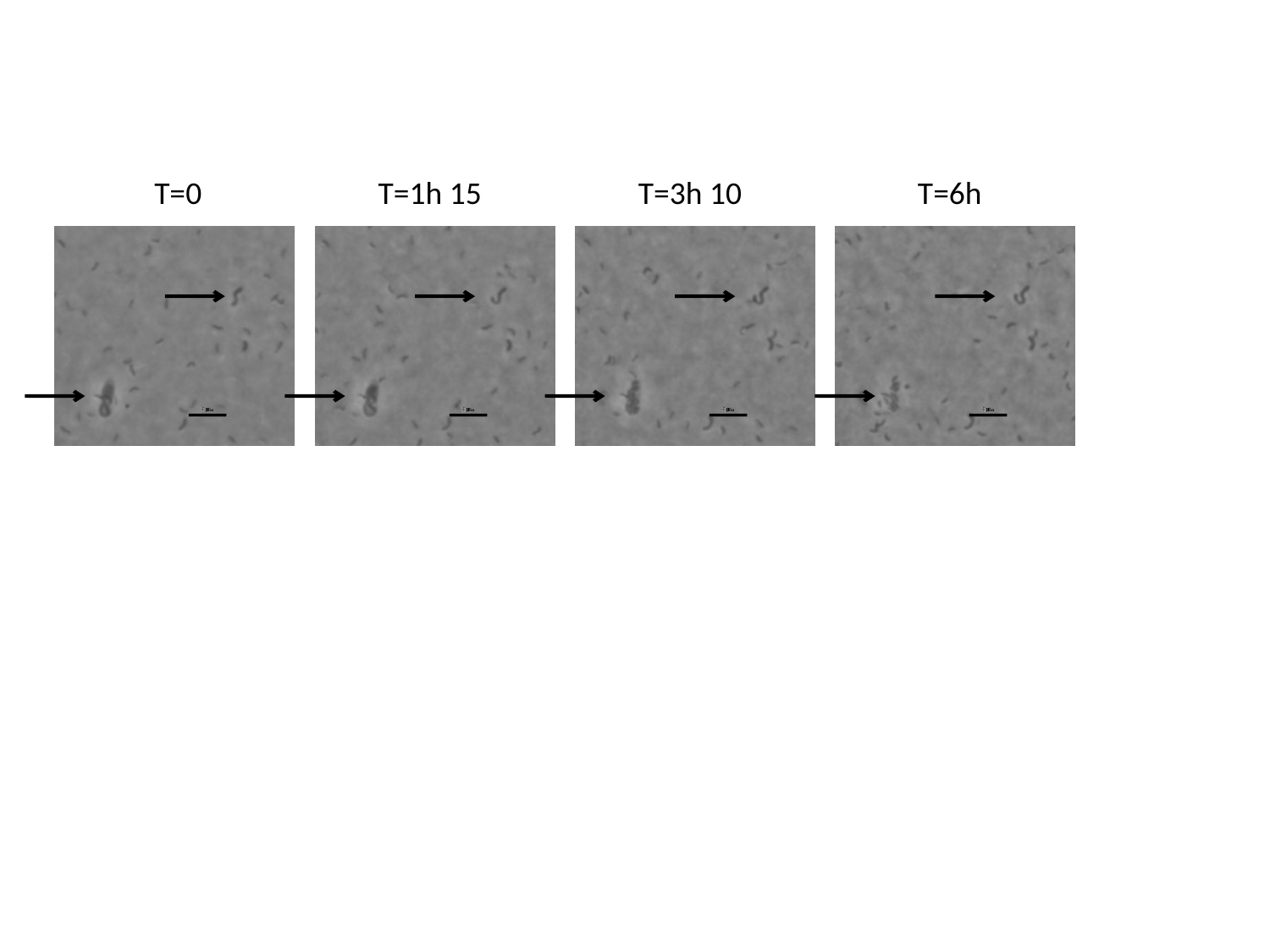

T=0
T=1h 15
T=3h 10
T=6h

Supplement: Additional file 2 — Stills from timelapse microscopy video showing simultaneous growth and division of B. bacteriovorus Tiberius: A filamentous B. bacteriovorus Tiberius grows, divides and fragments in a large E.coli prey bdelloplast (lower arrow) while a smaller B. bacteriovorus Tiberius (upper arrow) elongates prey-independently. Shown by time lapse microscopy from a predator-prey culture in calcium-HEPES buffer, supported on an agarose pad on a microscope slide. [file 1471-2164-13-670-S2.ppt]

## Slide 1
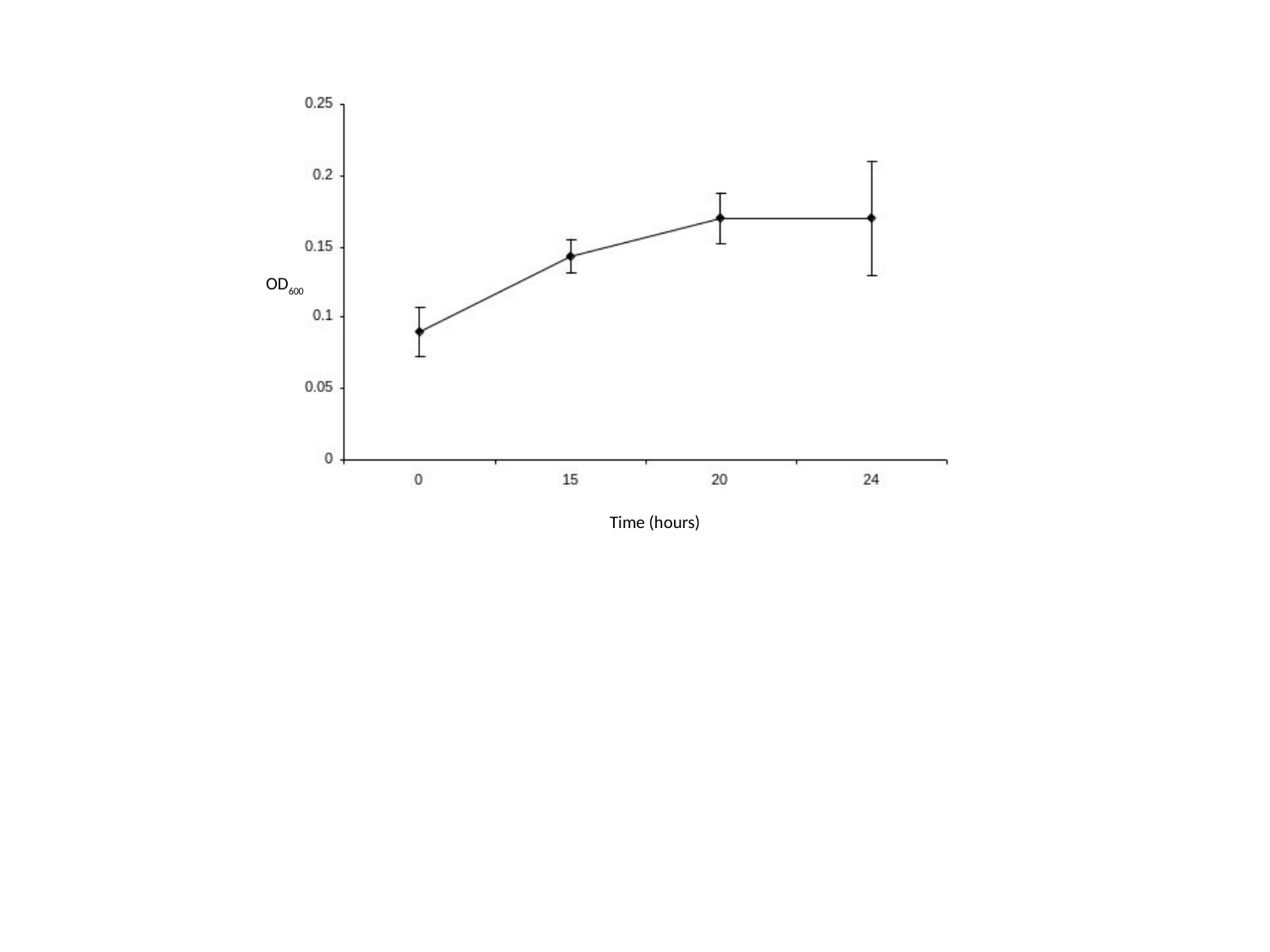

OD600
Time (hours)

Supplement: Additional file 3 — Graph showing B. bacteriovorus Tiberius host-independent growth of cells from a predatorily grown culture: Spontaneously streptomycin-resistant Tiberius cells were taken directly from a culture grown on prey cells. They were then directly grown in PY broth (Horowitz et al., 1974) in the presence of streptomycin to inhibit growth of prey E. coli (still present in the co-culture). The resulting Tiberius growth was measured as an increase in OD at 600 nm, shown in the graph against time of incubation at 29°C. The larger prey-independent cells of Tiberius were large enough to measure by optical density (although attack phase sized Bdellovibrio are not) , thus the OD600nm values are probably an under-estimate of the growth rate of total Bdellovibrio (including smaller cells) in the population. The data shown are from 3 independent repeats and the error bars are 1 standard deviation from the mean. [file 1471-2164-13-670-S3.ppt]

## Slide 1
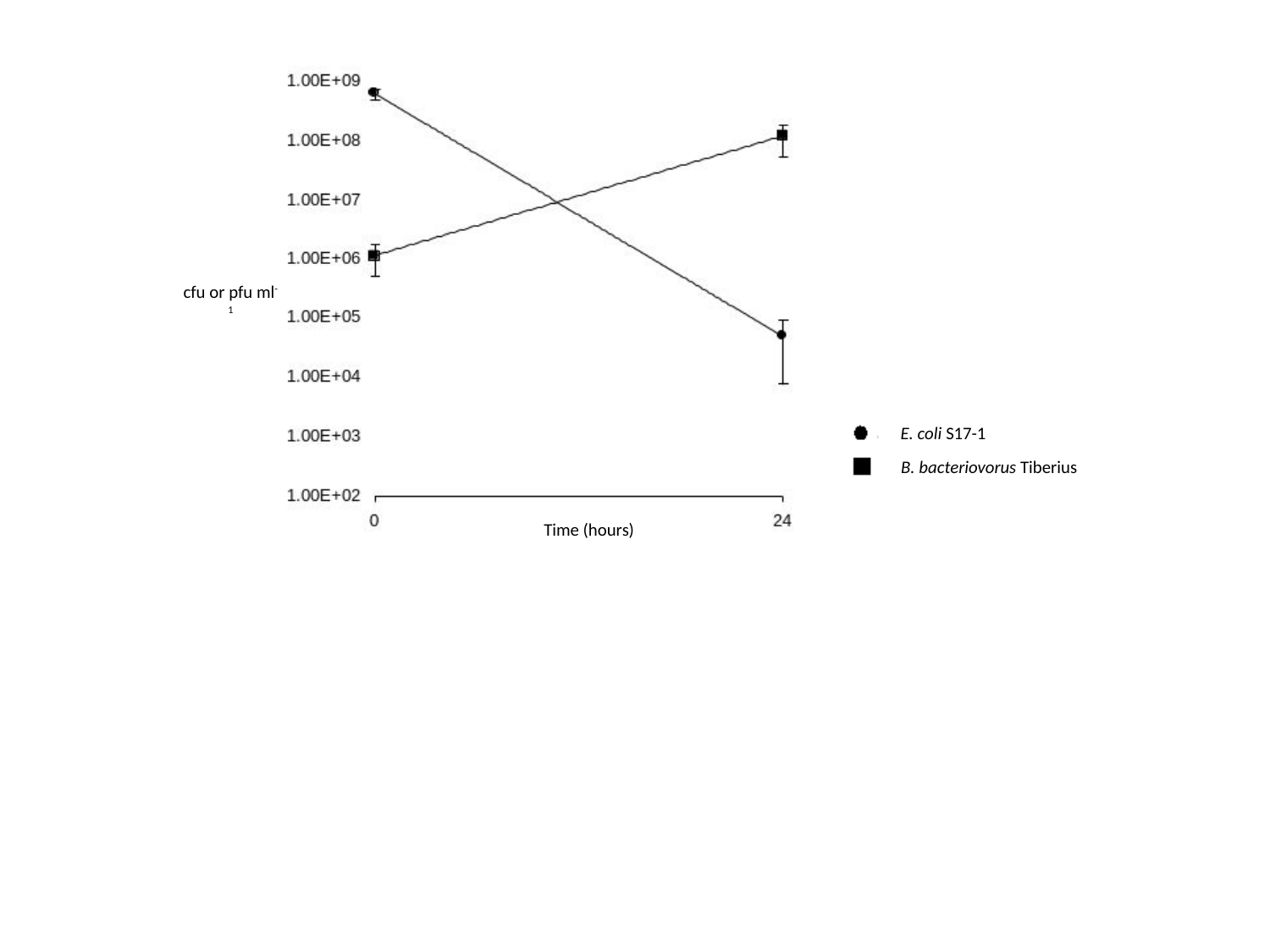

cfu or pfu ml-1
E. coli S17-1
B. bacteriovorus Tiberius
Time (hours)

Supplement: Additional file 4 — Graph showing evidence of killing of E. coli S17-1 and growth of B. bacteriovorus Tiberius in a predatory culture. Start- and end-point analyses of viable cell numbers of E. coli prey cells and Tiberius predatory cells by plaque (Tiberius) and colony (E. coli) counts on soft agar overlays on prey lawns, or conventional LB agar plates, respectively. [file 1471-2164-13-670-S4.ppt]

## Slide 1
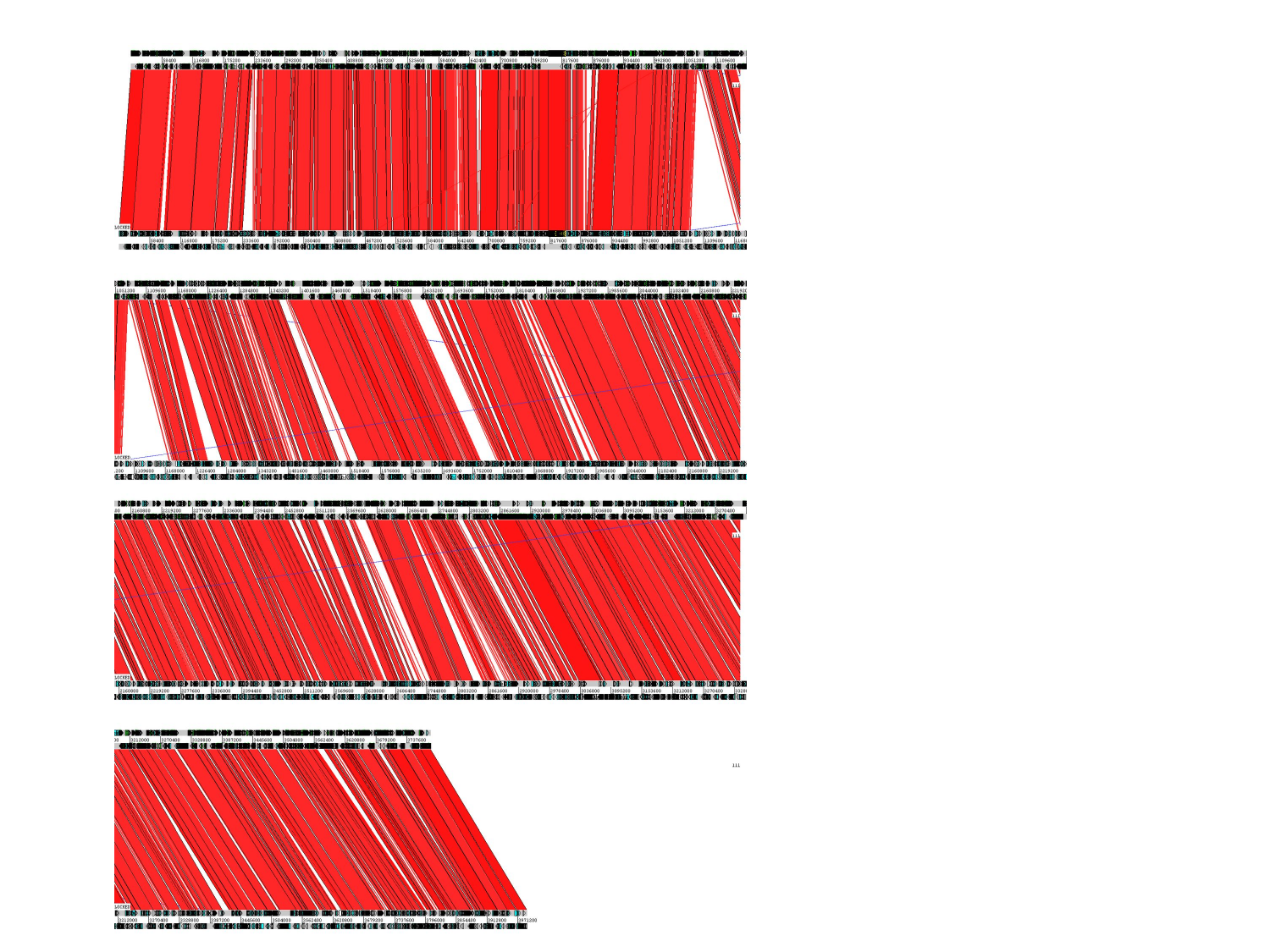

Supplement: Additional file 5 — ACT comparison of B. bacteriovorus HD100 and Tiberius genomes. HD100 is shown along the top of the comparison, with Tiberius below. Areas shown in red are regions of high synteny; blue lines represent areas of homology but in reverse orientation. [file 1471-2164-13-670-S5.ppt]
