# Supplementary material for: Genome analysis of a simultaneously predatory and prey-independent, novel Bdellovibrio bacteriovorus from the River Tiber, supports in silico predictions of both ancient and recent lateral gene transfer from diverse bacteria
Source: BMC Genomics. 2012 Nov 27;13:670. doi: 10.1186/1471-2164-13-670 (PMC3539863; doi:10.1186/1471-2164-13-670)
Supplement: Additional file 8 — Table showing the location and composition of unique gene islands located next to tRNAs. [file 1471-2164-13-670-S8.doc]

Unique Gene Islands and Phage genes at tRNAs in each Strain

| Unique Gene Islands at tRNAs in Tiberius | # of genes | Location | Includes |
| --- | --- | --- | --- |
| Bdt228-244 | 16 | tRNA bdt227 | Recombinase, hypothetical proteins, restriction endonuclease genes |
| Bdt1187-1202 | 11 | tRNA bdt1186 | Phage integrase, hypothetical proteins, IS2 element transposase insD gene |
| Bdt1444-1452 | 9 | tRNA bdt1453 | Resolvase, hypothetical proteins, IS2 element transposase insD genes |
| Bdt2587-2601 | 14 | tRNA bdt2603 | Glutathione S transferase , helicase and hypothetical proteins, two copies of IS2 element transposase insD genes |

| Unique Gene Islands at tRNAs in HD100 | # of genes | Location | Includes |
| --- | --- | --- | --- |
| Bd0090-0093 | 4 | tRNA001 | Hypothetical proteins, cytochrome b gene, 2 internal tRNAs (tRNA002 and tRNA003) |
| Bd0243-0251 | 5 | tRNA005 |  |
| Bd0929-0933 | 5 | tRNA0011 | merR family regulator, protease and chemotaxis sensor genes |
| Bd1743-1745 | 2 | tRNA0018 | Hypothetical proteins, cysteine synthase gene, *lysR* regulator |
| Bd2296-2298 | 3 | tRNA0026 | Cation transporting ATPase, hypothetical proteins |
| Bd2666-2682 | 16 | tRNA0027 | Protease, reverse transcriptase, nucleotide binding protein, cytochrome c, hypothetical proteins. tRNA0027 is the site of the 2^nd^ largest island insertion (of a different set of genes) in the Tiberius genome (bdt2603). |
| Bd2898-2899 | 2 | tRNA0029 | Hypothetical proteins and *hemG* |
| Bd2802-2803 | 2 | tRNA0028 | Hypothetical proteins |
| Bd2261-2262 | 2 | tRNA0025 | Hypothetical proteins |
| Bd2133-2134 | 2 | tRNA0024 | Hypothetical proteins and phage tail fibre gene |
| Bd1458-1459 | 2 | tRNA0014 | Hypothetical proteins |
