# Supplementary material for: Genome analysis of a simultaneously predatory and prey-independent, novel Bdellovibrio bacteriovorus from the River Tiber, supports in silico predictions of both ancient and recent lateral gene transfer from diverse bacteria
Source: BMC Genomics. 2012 Nov 27;13:670. doi: 10.1186/1471-2164-13-670 (PMC3539863; doi:10.1186/1471-2164-13-670)
Supplement: Additional file 9 — a. DNA encoding ICE like transposase element, 6 identical insertions of which were unique to the B. bacteriovorus Tiberius genome.b. predicted domains on encoded protein from Blast X search. c. Alignment of Tiberius encoded IS element product translated from bases 7–732 with insertion element IS2 transposase catalytic protein InsD of E.coli. The amino-acid sequences are 48% identical. [file 1471-2164-13-670-S9.ppt]

## Slide 1
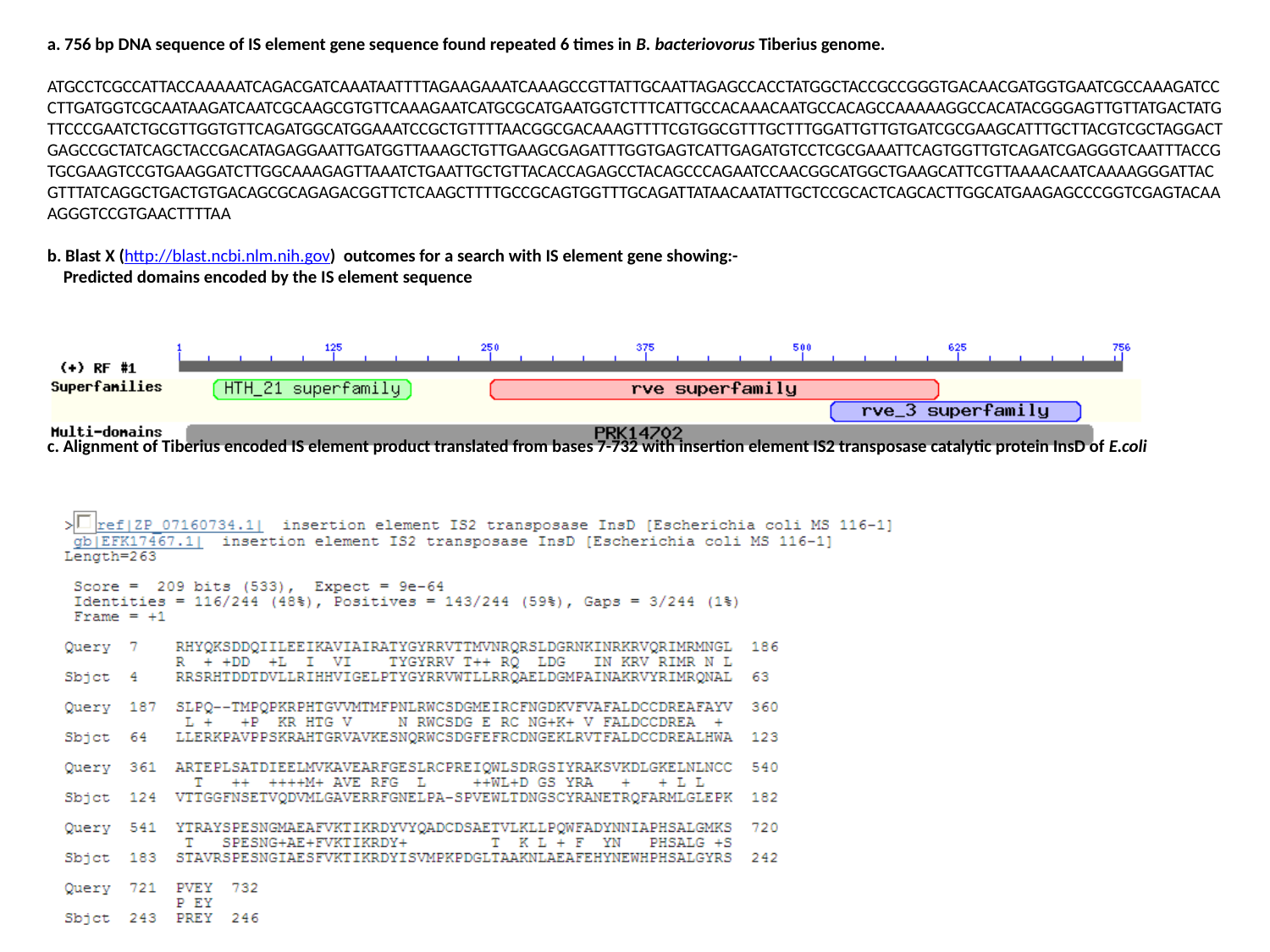

a. 756 bp DNA sequence of IS element gene sequence found repeated 6 times in B. bacteriovorus Tiberius genome.
ATGCCTCGCCATTACCAAAAATCAGACGATCAAATAATTTTAGAAGAAATCAAAGCCGTTATTGCAATTAGAGCCACCTATGGCTACCGCCGGGTGACAACGATGGTGAATCGCCAAAGATCCCTTGATGGTCGCAATAAGATCAATCGCAAGCGTGTTCAAAGAATCATGCGCATGAATGGTCTTTCATTGCCACAAACAATGCCACAGCCAAAAAGGCCACATACGGGAGTTGTTATGACTATGTTCCCGAATCTGCGTTGGTGTTCAGATGGCATGGAAATCCGCTGTTTTAACGGCGACAAAGTTTTCGTGGCGTTTGCTTTGGATTGTTGTGATCGCGAAGCATTTGCTTACGTCGCTAGGACTGAGCCGCTATCAGCTACCGACATAGAGGAATTGATGGTTAAAGCTGTTGAAGCGAGATTTGGTGAGTCATTGAGATGTCCTCGCGAAATTCAGTGGTTGTCAGATCGAGGGTCAATTTACCGTGCGAAGTCCGTGAAGGATCTTGGCAAAGAGTTAAATCTGAATTGCTGTTACACCAGAGCCTACAGCCCAGAATCCAACGGCATGGCTGAAGCATTCGTTAAAACAATCAAAAGGGATTACGTTTATCAGGCTGACTGTGACAGCGCAGAGACGGTTCTCAAGCTTTTGCCGCAGTGGTTTGCAGATTATAACAATATTGCTCCGCACTCAGCACTTGGCATGAAGAGCCCGGTCGAGTACAAAGGGTCCGTGAACTTTTAA
b. Blast X (http://blast.ncbi.nlm.nih.gov) outcomes for a search with IS element gene showing:-
 Predicted domains encoded by the IS element sequence
c. Alignment of Tiberius encoded IS element product translated from bases 7-732 with insertion element IS2 transposase catalytic protein InsD of E.coli
