# Supplementary material for: Genome analysis of a simultaneously predatory and prey-independent, novel Bdellovibrio bacteriovorus from the River Tiber, supports in silico predictions of both ancient and recent lateral gene transfer from diverse bacteria
Source: BMC Genomics. 2012 Nov 27;13:670. doi: 10.1186/1471-2164-13-670 (PMC3539863; doi:10.1186/1471-2164-13-670)
Supplement: Additional file 10 — Table showing the location and type of each tRNA identified by tRNAScan-SE in the genomes of B. bacteriovorus HD100 and Tiberius. Note the absence of the Pro-type tRNA from the Tiberius genome. [file 1471-2164-13-670-S10.doc]

| HD100 | | | | tiberius | | | |
| --- | --- | --- | --- | --- | --- | --- | --- |
| locus_tag | Start bp | Stop bp | Type | locus_tag | Start bp | Stop bp | Type |
| trna_0001 | 73069 | 73158 | Ser | Bdt0073 | 73051 | 73140 | Ser |
| trna_0002 | 86076 | 85986 | Ser | Bdt0088 | 89220 | 89130 | Ser |
| trna_0003 | 86219 | 86295 | Arg | Bdt0089 | 89363 | 89439 | Arg |
| trna_0004 | 88640 | 88715 | Asn | Bdt0091 | 90021 | 90096 | Asn |
| trna_0005 | 229983 | 230059 | Met | Bdt0227 | 233725 | 233801 | Met |
| trna_0006 | 313766 | 313676 | Ser | Bdt0326 | 332718 | 332628 | Ser |
| trna_0007 | 632668 | 632586 | Leu | Bdt0652 | 658717 | 658635 | Leu |
| trna_0008 | 761752 | 761827 | Ala | Bdt0771 | 782489 | 782564 | Ala |
| trna_0009 | 819230 | 819305 | Ala | Bdt0831 | 842045 | 842120 | Ala |
| trna_0010 | 821232 | 821308 | Ile | Bdt0833 | 844037 | 844113 | Ile |
| trna_0011 | 888386 | 888310 | Pro | Bdt0885 | 898052 | 897976 | Pro |
| trna_0012 | 1151153 | 1151228 | Arg | Bdt1186 | 1249241 | 1249317 | Arg |
| trna_0013 | 1274601 | 1274677 | Asp | Bdt1323 | 1398731 | 1398807 | Asp |
| trna_0014 | 1380844 | 1380769 | Glu | Bdt1453 | 1535191 | 1535116 | Glu |
| trna_0015 | 1380947 | 1380872 | Lys | Bdt1454 | 1535294 | 1535219 | Lys |
| trna_0016 | 1465861 | 1465780 | Leu | Bdt1535 | 1623177 | 1623096 | Leu |
| trna_0017 | 1576730 | 1576806 | Pro |  |  |  |  |
| trna_0018 | 1675205 | 1675281 | His | Bdt1717 | 1826722 | 1826797 | His |
| trna_0019 | 1689296 | 1689372 | Ile | Bdt1731 | 1839305 | 1839381 | Ile |
| trna_0020 | 1720462 | 1720371 | Leu | Bdt1771 | 1869914 | 1869823 | Leu |
| trna_0021 | 1805359 | 1805434 | Gly | Bdt1849 | 1955407 | 1955482 | Gly |
| trna_0022 | 1809285 | 1809371 | Leu | Bdt1855 | 1959802 | 1959888 | Leu |
| trna_0023 | 1974011 | 1974086 | Val | Bdt2030 | 2130115 | 2130190 | Val |
| trna_0024 | 2038362 | 2038438 | Met | Bdt2093 | 2197814 | 2197890 | Met |
| trna_0025 | 2159706 | 2159624 | Leu | Bdt2216 | 2321132 | 2321050 | Leu |
| trna_0026 | 2193953 | 2193880 | Cys | Bdt2251 | 2356450 | 2356377 | Cys |
| trna_0027 | 2606187 | 2606111 | Met | Bdt2602 | 2752468 | 2752392 | Met |
| trna_0028 | 2712668 | 2712743 | Gln | Bdt2727 | 2876639 | 2876714 | Gln |
| trna_0029 | 2810194 | 2810118 | Arg | Bdt2838 | 2996431 | 2996355 | Arg |
| trna_0030 | 2890262 | 2890187 | Trp | Bdt2920 | 3076304 | 3076229 | Trp |
| trna_0031 | 2891786 | 2891712 | Thr | Bdt2922 | 3077871 | 3077797 | Thr |
| trna_0032 | 2891878 | 2891802 | Gly | Bdt2923 | 3077963 | 3077887 | Gly |
| trna_0033 | 2892036 | 2891951 | Tyr | Bdt2924 | 3078120 | 3078035 | Tyr |
| trna_0034 | 2892139 | 2892064 | Thr | Bdt2925 | 3078223 | 3078148 | Thr |
| trna_0035 | 3615999 | 3615924 | Val | Bdt3627 | 3833444 | 3833369 | Val |
| trna_0036 | 3666784 | 3666709 | Phe | Bdt3673 | 3880332 | 3880257 | Phe |
